# Systemic Treatment with CpG-B after Sublethal Rickettsial Infection Induces Mouse Death through Indoleamine 2,3-Dioxygenase (IDO)

**DOI:** 10.1371/journal.pone.0034062

**Published:** 2012-03-28

**Authors:** Lijun Xin, Thomas R. Shelite, Bin Gong, Nicole L. Mendell, Lynn Soong, Rong Fang, David H. Walker

**Affiliations:** 1 Department of Pathology and Center for Biodefense and Emerging Infectious Diseases, University of Texas Medical Branch, Galveston, Texas, United States of America; 2 Department of Microbiology and Immunology, University of Texas Medical Branch, Galveston, Texas, United States of America; National Jewish Health and University of Colorado School of Medicine, United States of America

## Abstract

Due to its strong immune stimulatory effects through TLR9, CpG-containing oligodeoxynucleotides (CpG ODN) have been tested in multiple clinical trials as vaccine adjuvant for infectious diseases and cancer. However, immune suppression induced by systemic administration of CpGs has been reported recently. In this study, we evaluated the impact of CpGs in an acute rickettsiosis model. We found that systemic treatment with type B CpG (CpG-B), but not type A CpG (CpG-A), at 2 days after sublethal *R. australis* infection induced mouse death. Although wild-type (WT) B6 and IDO^−/−^ mice showed similar survival rates with three different doses of *R. australis* infection, treatment with CpG-B after sublethal infection consistently induced higher mortality with greater tissue bacterial loads in WT but not IDO^−/−^ mice. Also, CpG-B treatment promoted the development of higher serum concentrations of proinflammatory cytokines/chemokines through IDO. Furthermore, while T cell-mediated immune responses enhanced by CpG-B were independent of IDO, treatment with CpG-B promoted T cell activation, PD-1 expression and cell apoptosis partially through IDO. A depletion study using anti-mPDCA-1 mAb indicated that plasmacytoid dendritic cells (pDC) were not required for CpG-B-induced death of *R. australis*-infected mice. Additionally, the results in iNOS^−/−^ mice suggested that nitric oxide (NO) was partially involved in CpG-B-induced death of *R. australis*-infected mice. Surprisingly, pre-treatment with CpG-B before administration of a lethal dose of *R. australis* provided effective immunity in WT, IDO^−/−^ and iNOS^−/−^ mice. Taken together, our study provides evidence that CpGs exert complex immunological effects by both IDO-dependent and -independent mechanisms, and that systemic treatment with CpGs before or after infection has a significant and distinct impact on disease outcomes.

## Introduction

TLR9 is expressed intracellularly in various cells including B cells, monocytes, macrophages, NK cells, dendritic cells (DCs), and plasmacytoid dendritic cells (pDCs). Activation of TLR9 by its ligand, CpG ODN, induces a predominant Th1 innate and adaptive immune response, including secretion of pro-inflammatory cytokines, and IFN-inducible cytokines/chemokines, induction of IgG isotype switching and antibody secretion, differentiation of Th1 cells, and enhancement of NK and CTL functional activities [1], [2]. Previous studies have extensively shown that pretreatment with CpGs provides solid protection against various infectious diseases [3], [4], [5], [6] and immunotherapy for cancer and allergy [7], [8]. Currently, synthetic CpGs are being tested in multiple clinical trials as adjuvants for vaccines against infectious diseases and cancer [9], [10].

As the first and rate-limiting enzyme in the catabolism of tryptophan, indoleamine 2,3-dioxygenase (IDO) has been recently documented to play an immunosuppressive role in peripheral tolerance in pregnancy, infection, transplantation, autoimmunity, allergy, and cancer [11], [12]. A recent study of experimental asthma has demonstrated that systemic treatment with CpGs induces strong pulmonary IDO activity to inhibit Th-mediated lung inflammation [13]. Further studies also showed that CpGs exert an immune suppressive effect by induction of IDO. It has been reported that systemic administration of CpGs to mice induces rare CD19^+^ DCs in spleen to express IDO and to acquire potent T cell suppressor activity through costimulation of CTLA-4 and/or PD-1 [14], [15]. Similarly, compared to T cell activation induced by subcutaneous administration of CpGs, systemic treatment with CpGs suppresses T cell expansion and CTL activity via induction of IDO [16]. Taken together, CpGs may exert either immune stimulatory or suppressive functions, depending on the route of application, such as subcutaneous versus intravenous or intraperitoneal administration.

In the present study, we further evaluated the immunological effects of CpGs in a mouse model of an acute infectious disease, rickettsiosis, which is an emerging/re-emerging infectious disease caused by tick-borne pathogens of the genus *Rickettsia* [17], [18], [19]. We found that, although pre-treatment with CpG-B provided complete immunity against an ordinarily lethal dose of *R. australis*, systemic treatment with CpG-B after a sublethal dose of *R. australis* induced death of the animals. Our results indicate that IDO is involved in CpG-B-mediated immune responses and the timing of CpG treatment affects the infectious disease outcome.

## Methods

### Mice

C57BL/6J (B6), IDO^−/−^ (IDO1-deficient) and iNOS^−/−^ mice on B6 background were purchased from the Jackson Laboratory (Bar Harbor, ME). IDO^−/−^ mice were bred and housed in the University Animal Facility. Sex- and gender-matched, 8–12 wk old mice were used in all studies. Experimental mice were housed in a biosafety level 3 facility, and all experiments and procedures were approved by the Institutional Animal Care and Use Committee of the University of Texas Medical Branch, Galveston.

### Rickettsial infection

*Rickettsia australis* (Cutlack strain) were passaged and maintained in embryonated chicken yolk sacs in our laboratory [17]. The 10% yolk sac suspension stock in SPG buffer (218 mM sucrose, 10 mM potassium phosphate, pH 7.0, 5 mM potassium glutamate) contained 4×10^7^ pfu per ml. After diluted in SPG buffer, the indicated dose of *R. australis* (200 µl per mouse) was injected i.v. through the tail vein. Control mice were inoculated with 200 µl of 10% yolk sac in SPG buffer. Mice were monitored daily for signs of illness until day 14.

### CpG ODNs

Type B CpG (CpG-B, ODN 1826 sequence: 5′-TCCATGACGTTCCTGACGTT-3′) and ODN 1826 control, Type A CpG (CpG-A, ODN 1585 sequence: 5′- GGGGTCAACGTTGAGGGGGG -3′) and ODN 1585 control were purchased from InvivoGen (San Diego, CA). For *in vivo* experiments, 50 µg of CpGs or CpG control per mouse were injected i.v. 2 days before or after *R. australis* infection.

### IDO enzymatic activity assay

Tissue IDO enzymatic activity was measured by a colorimetric method with minor modifications [20]. Briefly, spleen, lung, liver and brain tissues were collected from mice on day 5 post-infection and homogenized. After centrifugation, the tissue lysates (150 µl) were added into an equal amount of 2× IDO buffer (100 mM PBS, pH 6.5, 40 mM ascorbate, 20 µM methylene blue, 200 µg/ml catalase, and 800 mM L-tryptophan; all reagents were purchased from Sigma-Aldrich (St. Louis, MO) and incubated for 1 h at 37°C. The reaction was stopped with the addition of 50 µl of 30% trichloroacetic acid and further incubated for 30 min at 52°C. After centrifugation, supernatant was mixed with an equal amount of Ehrlich's reagent (2% *p*-dimethylaminobenzaldehyde in acetic acid). The color was allowed to develop for 10 min, and then the absorbance was measured at 490 nm in a spectrophotometer. Kynurenine (Kyn) concentration in the supernatant was calculated by an L-Kyn standard curve. The IDO activity was expressed as nmol Kyn formed/h/mg protein. The amount of protein in the samples was measured by bis-cinchonic acid (BCA) method with bovine serum albumin as standard.

### Quantification of rickettsial loads by real-time PCR

To determine the rickettsial loads in infected organs, mouse tissues including spleen, liver, lung and brain were collected on day 5 postinfection and homogenized. DNA was extracted using a DNeasy tissue kit (Qiagen, Valencia, CA), and rickettsial loads were determined using an iCycler IQ from Bio-Rad (Hercules, CA). The following primers and probes (Biosearch Technologies, Novato, CA) targeting *R. australis* citrate synthase (CS) gene (U59718.1) and mouse glyceraldehyde-3-phosphate dehydrogenase (GAPDH) gene were used as described previously [21]: CS forward primer 5′-GAGAGAAAATTATATCCAAATGTTGAT-3′, CS reverse primer 5′-AGGGTCTTCGTGCATTTCTT-3′, CS probe 5′-FAM-CATTGTGCCATCCAGCCTACGGT-BHQ-1-3′, GAPDH forward primer 5′-CAACTACATGGTCTACATGTTC-3′, GAPDH reverse primer 5′-CTCGCTCCTGGAAGATG-3′, and GAPDH probe 5′-FAM-CGGCACAGTCAAGGCCGAGAATGGGAAGC-BHQ-1-3′. The results of bacterial loads were normalized using GAPDH data for the same sample and expressed as the number of CS copies per 10^6^ copies of GAPDH.

### Flow cytometry

The following mAbs were purchased from BD Biosciences (San Jose, CA) or eBiosciences (San Diego, CA): fluorescein isothiocyanate (FITC)-conjugated anti-CD8 (53-6.7), anti-Ly-6C (AL-21), anti-IFN-γ (XMG1.2); phycoerythrin (PE)-conjugated anti-CD19 (1D3), anti-Ly-6G (1A8), anti-IL-10 (JES5-16E3), anti-TNF-α (MP6-XT22), anti-IL-17A (TC11-18H10), anti-PD-1 (J43); PerCP Cy5.5-conjugated anti-CD3 (145-2C11); PE-Cy7 anti-CD4 (RM4-5); allophycocyanin (APC)-conjugated anti-CD25 (PC61), anti-CD11b (M1/70), anti-CD8 (53-6.7); as well as isotype control Abs, including FITC-conjugated rat IgG1, PE-conjugated rat IgG1, IgG2b, and APC-conjugated rat IgG2a. PE-conjugated anti-mouse/rat Foxp3 (FJK-16s) staining reagents were purchased from eBiosciences.

At day 5 post-infection, spleens from individual mice were collected and processed. After red blood cells were lysed, single splenocytes were counted and stained for surface markers. Intracellular Foxp3 staining was performed following the manufacturer's protocol. For intracellular cytokine staining, splenocytes were restimulated with phorbol 12-myristate 13-acetate (PMA)/Ionomycin/GolgiStop (BD Biosciences) for 5 h. After T cell surface marker staining, intracellular cytokines (IFN-γ, IL-10, TNF-α, IL-17) were stained with antibodies by following cytofixation/permeabilization with a Cytofix/Cytoperm Kit (BD Biosciences). Samples were evaluated on a FACSCanto or LSRFortessa flow cytometer (BD Biosciences), and results were analyzed by using FlowJo software (TreeStar, Ashland, OR, USA).

### Measurement of serum cytokines by Bio-Plex

Sera were collected from mice at day 5 post-infection. Multiple serum cytokines and chemokines (IL-1β, IL-6, IL-10, IL-12p40, IFN-γ, TNF-α, MCP-1, and RANTES) were measured by Bio-Plex cytokine assay (Bio-Rad Laboratories, Hercules, CA). Serum IFN-γ levels were verified by Quantikine ELISA kit (R&D Systems, Minneapolis, MN).

### Tissue histology and TUNEL assay

On day 5 post-infection, liver and spleen tissues were collected, fixed with 10% neutral buffered formalin, and embedded in paraffin. Tissue sections (5 µm thickness) were stained with hematoxylin and eosin. TUNEL assay was performed on formalin-fixed, paraffin-embedded liver and spleen tissues by using *in situ* cell death detection kit (fluorescein) according to the manufacturer's instructions (Roche Diagnostics, Indianapolis, IN).

### Plasmacytoid dendritic cell depletion

Functional grade of anti-mouse PDCA-1 (JF05-1C2.4.1) and PE-conjugated anti-mPDCA-1 mAbs were purchased from Miltenyi Biotec (Auburn, CA). To deplete plasmacytoid dendritic cells (pDCs) *in vivo*, C57BL/6J mice were injected i.v. with 500 µg of anti-mPDCA-1 mAb or control rat IgG one day before *R. australis* infection, then four times i.p. with 250 µg of mAb or rat IgG at one hour before, and 2, 4, 6 days after i.v. infection with 5×10^5^ pfu of *R. australis*. Then, 50 µg of CpG-B per mouse were injected i.v. on day 2 post-infection. Survival of mice was monitored for 14 days. At day 7, splenocytes from control and pDC-depleted mice were stained with FITC-conjugated anti-Ly-6C and PE-conjugated anti-mPDCA-1. The efficiency of pDC depletion was 80–90%.

### Statistical analyses

Numeric data were presented as means ± standard deviation. Statistical analyses for survival curves were calculated by the log-rank test, and the comparison between two different groups was determined by a two-tailed Mann-Whitney test using GraphPad Prism, version 5.00, for Windows (GraphPad Software, San Diego, CA). Statistically significant values are referred to as follows: *, *p*<0.05; **, *p*<0.01.

## Results

### Systemic treatment with CpG-B, but not CpG-A, after sublethal *R. australis* infection induced death of mice

**Figure 1:**
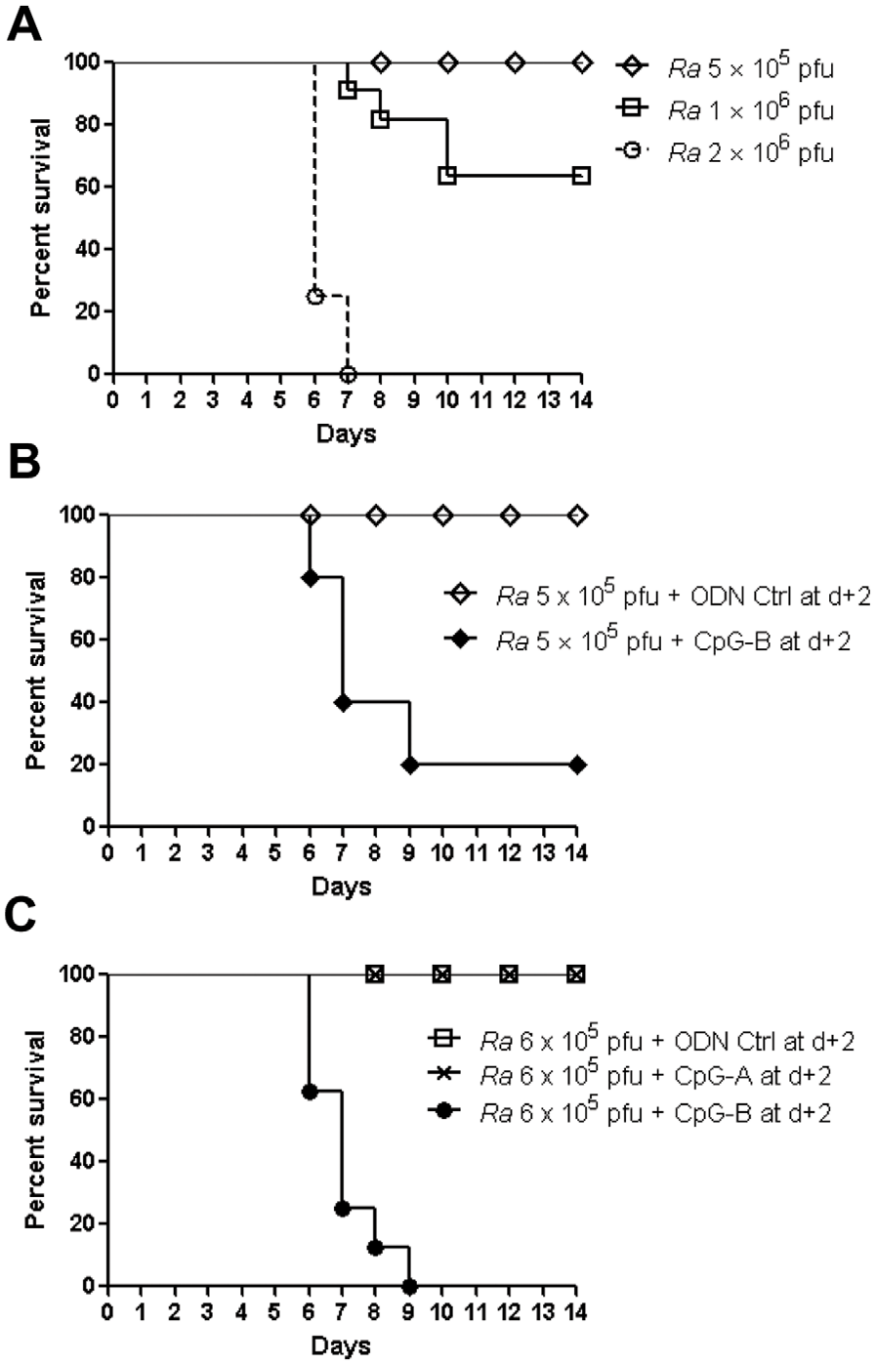
CpG-B, not CpG-A, treatment induces mortality in otherwise sublethal rickettsial infection. B6 mice (8 mice per group) were injected i.v. with different doses of *R. australis* (*Ra*) (A). At day 2 after infection with 5×10^5^ pfu of *R. australis*, 50 µg of ODN control (ODN 1826 control) and CpG-B (ODN 1826) per mouse were injected i.v., respectively (B). At day 2 after B6 mice (8 mice per group) were injected i.v. with *R. australis* (6×10^5^ pfu), 50 µg of ODN control (ODN 1585 control), CpG-A (ODN 1585), and CpG-B (ODN 1826) per mouse were injected i.v., respectively (C). Survival was monitored for 14 days.

By using the synthesized CpG ODNs, we investigated the immunological effects of a TLR9 agonist on rickettsial infection. As demonstrated in our previous study [17], infection with *R. australis* induced disease in C57BL/6J mice. All mice survived 5×10^5^ pfu, all died after infection with 2×10^6^ pfu, and half survived infection with 1×10^6^ pfu (Figure 1A). Interestingly, we found that, systemic treatment with CpG-B (50 µg of CpG-B per mouse, i.v.) at 2 days after sublethal *R. australis* infection (5×10^5^ pfu/mouse) induced mortality in mice (Figure 1B). Mice infected with a lethal dose of *R. australis* (2×10^6^ pfu/mouse) died very similarly with or without post-infection treatment with CpG-B (data not shown). In contrast to predominant induction of type I IFNs by CpG-A, CpG-B is known to induce strong NF-κB signaling and B cell activation but only weak type I IFN secretion [1]. We confirmed that treatment with CpG-B, but not CpG-A, at 2 days after sublethal *R. australis* infection induced death of mice (Figure 1C). This result suggests that activation of NF-κB signaling by CpG-B rather than induction of type I IFN by CpG-A causes death of *R. australis*-infected mice.

### CpG-B induced death of *R. australis*-infected mice through IDO

**Figure 2:**
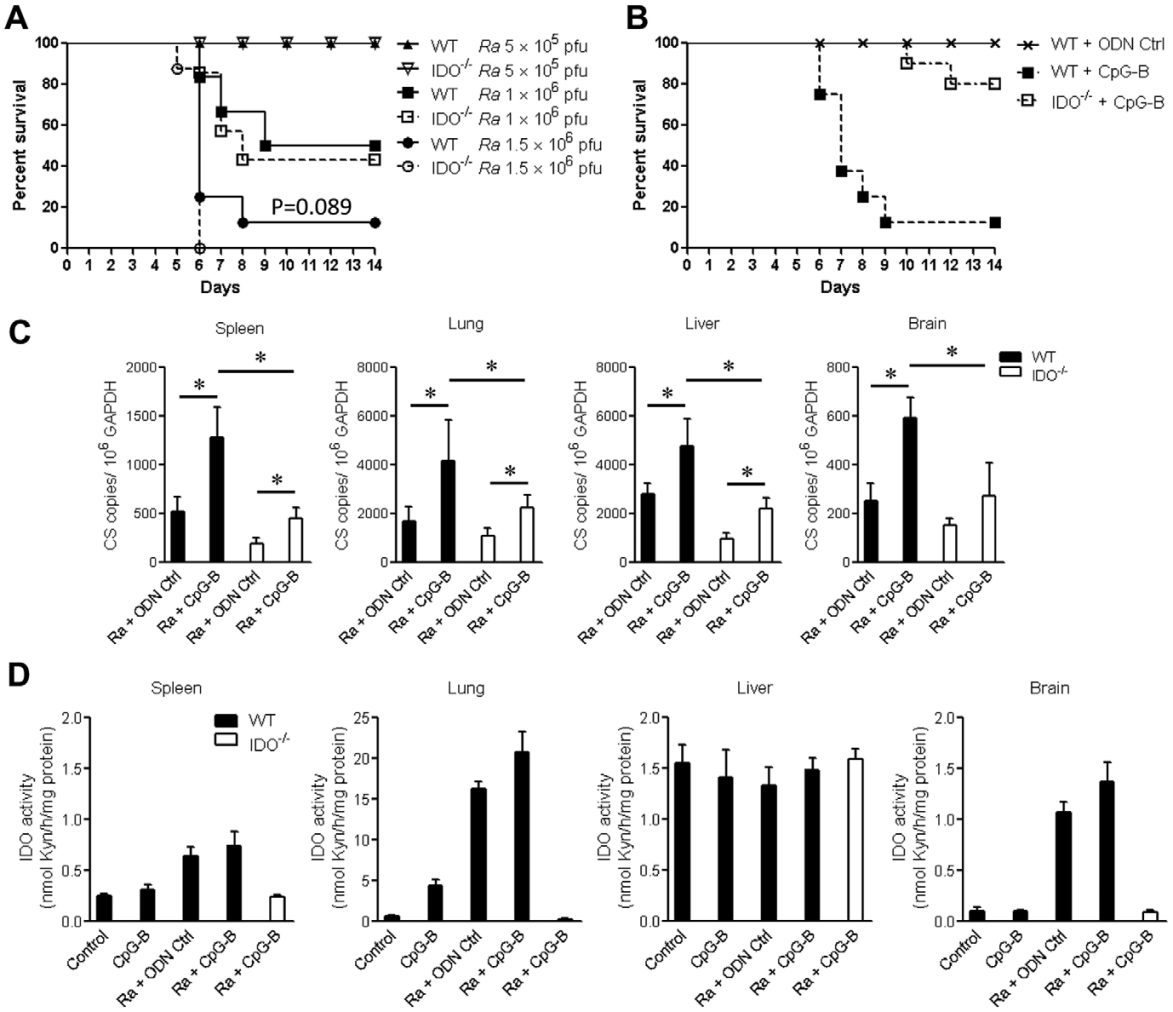
CpG-B induced death of *R. australis*-infected mice through IDO. WT and IDO^−/−^ mice (6–8 mice per group) were injected i.v. with different doses of *R. australis*. Mouse survival was monitored for 14 days (A). 50 µg of ODN control (ODN 1826 control) or CpG-B (ODN 1826) per mouse were injected i.v. into B6 and IDO^−/−^ mice (8 mice per group) at day 2 after infection with 5×10^5^ pfu of *R. australis*, respectively. Mouse survival was monitored for 14 days (B). Tissue bacterial loads determined by realtime PCR (C) and tissue IDO enzymatic activity determined by modified colorimetric assay (4 mice per group) were measured on day 5 post-infection (D). The representative results are shown as mean ± SD from three independent experiments. * *p*<0.05.

Previous studies have reported that systemic treatment with CpGs induces strong IDO expression in tissues [13], [16]. We wished to determine whether the IDO pathway is involved in CpG-induced death in *R. australis*-infected mice. By using IDO^−/−^ mice, we initially found that WT and IDO^−/−^ mice had comparable survival rates at three different doses of *R. australis* infection (Figure 2A), although *R. australis* infection did induce strong tissue expression of IDO in WT mice (Figure 2D). These results suggested a minor role of IDO in *R. australis* infection. However, we found that, when 50 µg of CpG-B per mouse was injected i.v. into WT and IDO^−/−^ mice 2 days after sublethal *R. australis* infection, 80–90% of IDO^−/−^ mice survived compared to the high mortality in WT mice (Figure 2B). At day 5 post-infection, treatment with CpG-B resulted in significantly increased tissue bacterial loads in infected WT and IDO^−/−^ mice compared to controls. However, tissue bacterial loads were significantly higher in CpG-B-treated, *R. australis*-infected WT mice than those in CpG-B-treated, *R. australis*-infected IDO^−/−^ mice (Figure 2C). We also confirmed that CpG alone and *R. australis* infection with or without CpG-B treatment induced strong IDO enzymatic activity in WT mouse tissues (especially in the lung) but not in IDO^−/−^ mouse tissues (Figure 2D). Tryptophan 2,3-dioxygenase (TDO) is constitutively expressed in liver, and the assay for IDO enzymatic activity does not distinguish IDO and TDO from one another in this organ. These results indicate that IDO plays a critical role in CpG-B-induced mouse death in rickettsial infection.

### CpG-B promoted different profiles of serum proinflammatory cytokines/chemokines

**Figure 3:**
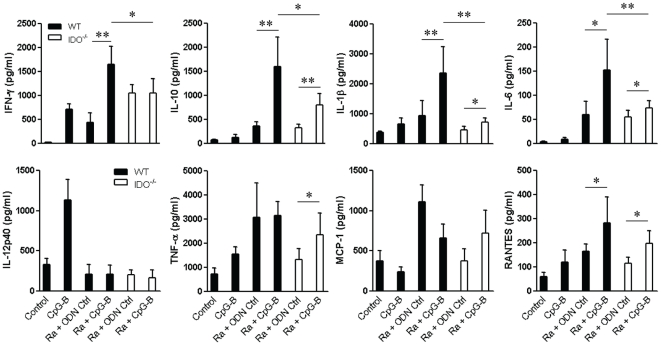
IDO is involved in the production of CpG-B-mediated-proinflammatory cytokines/chemokines. At day 2 after infection with 5×10^5^ pfu of *R. australis*, 50 µg of ODN control (ODN 1826 control) and CpG-B (ODN 1826) per mouse were injected i.v. into WT and IDO^−/−^ mice (4 mice per group). Mouse sera (6–8 mice per group) were collected at day 5 post-infection, multiple cytokines/chemokines were measured by Bio-plex assay and serum IFN-γ levels were verified by ELISA. * *p*<0.05 and ** *p*<0.01.

Using the Bio-Plex assay, we measured the levels of multiple proinflammatory cytokines/chemokines in mouse sera at day 5 post-infection. We found that treatment with CpG-B significantly enhanced the production of IFN-γ, IL-10, IL-1β, IL-6, and RANTES in *R. australis*-infected WT mice, compared to the only slightly enhanced production of IL-10, IL-1β, IL-6, TNF-α, and RANTES in CpG-B-treated, *R. australis*-infected IDO^−/−^ mice (Figure 3). Clearly, the serum levels of multiple cytokines (IFN-γ, IL-10, IL-1β, and IL-6) in CpG-B-treated, *R. australis*-infected WT mice were significantly higher than those in CpG-B-treated, *R. australis*-infected IDO^−/−^ mice (Figure 3). Interestingly, infection with *R. australis* induced little production of IL-12p40 and IL-17 (data not shown) *in vivo*. These results indicate that CpG-B differentially promotes the production of serum proinflammatory cytokines/chemokines during rickettsial infection.

### T cell-mediated immune response enhanced by CpG-B in *R. australis*-infected mice is IDO-independent

**Figure 4:**
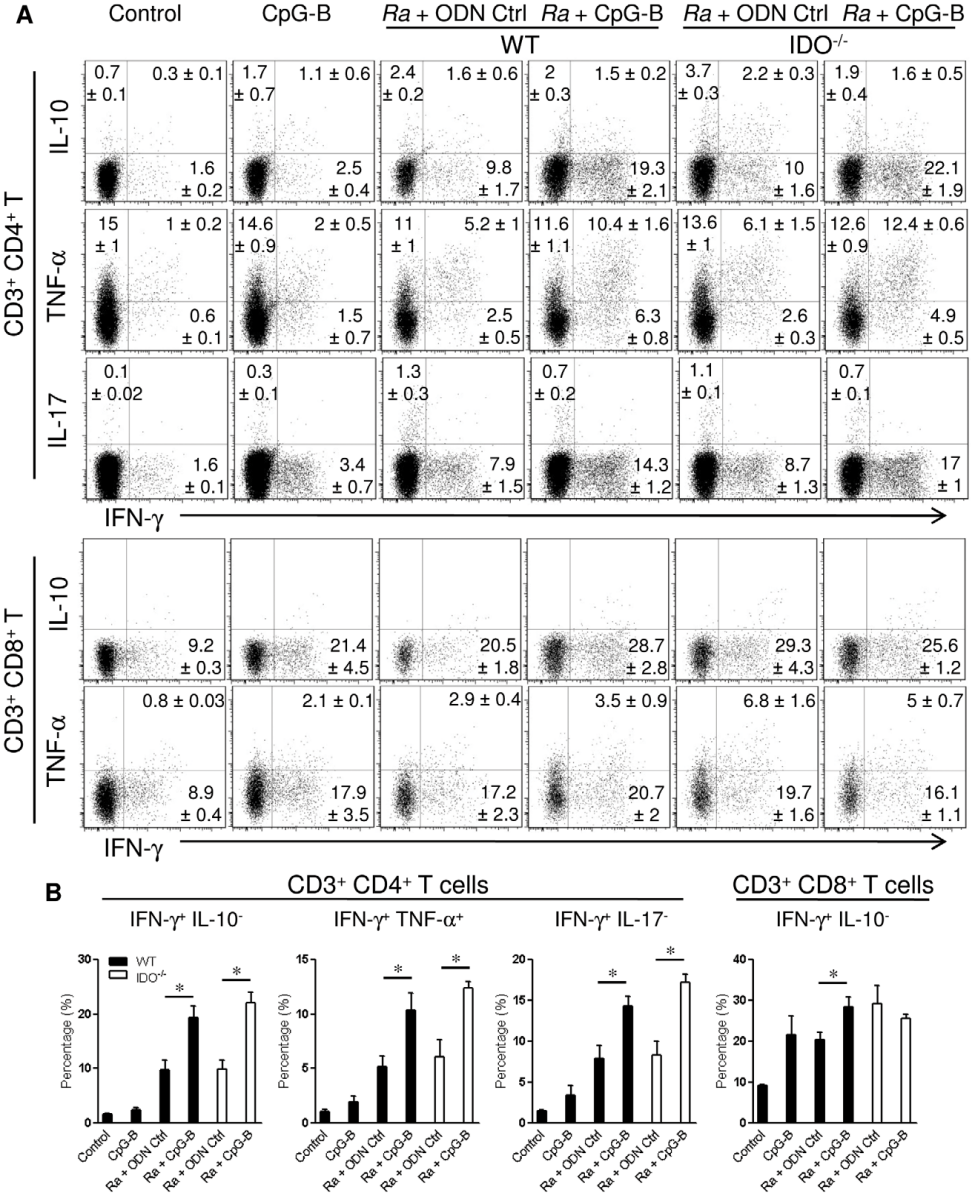
Multiple intracellular cytokines produced by CD4^+^ and CD8^+^ T cells. At day 2 after infection with 5×10^5^ pfu of *R. australis*, 50 µg of ODN control (ODN 1826 control) and CpG-B (ODN 1826) per mouse were injected i.v. into WT and IDO^−/−^ mice (4 mice per group). At day 5 post-infection, spleen cells (1×10^6^/ml) from individual mice were restimulated with PMA/Ionomycin/GolgiStop for 5 h. Multiple intracellular cytokines as indicated were measured in CD4^+^ and CD8^+^ T cells. The percentages of intracellular cytokine production in gated T cells were shown as mean ± SD in the corner (A). Representative statistical data are shown from one of three independent experiments. * *p*<0.05 (B).

To analyze T cell-mediated immune responses, spleen cells at day 5 post-infection were collected and restimulated with PMA/Ionomycin/GolgiStop *ex vivo*. We found that, *R. australis* infection induced comparable intracellular cytokine production in CD4^+^ T cells from WT and IDO^−/−^ mice. Treatment with CpG-B significantly increased cytokine production, especially IFN-γ and TNF-α, in CD4^+^ T cells from both WT and IDO^−/−^, *R. australis*-infected mice compared to the controls (Figure 4). While CpG-B significantly increased IFN-γ production in CD8^+^ T cells of infected WT mice, treatment with CpG-B did not affect IFN-γ production in CD8^+^ T cells from infected IDO^−/−^ mice (Figure 4). Taken together, these results indicate that treatment with CpG-B enhanced some T cell-mediated immune responses in an IDO-independent manner.

### CpG-B promoted T cell activation and PD-1 expression rather than increasing Treg cells during *R. australis* infection

**Figure 5:**
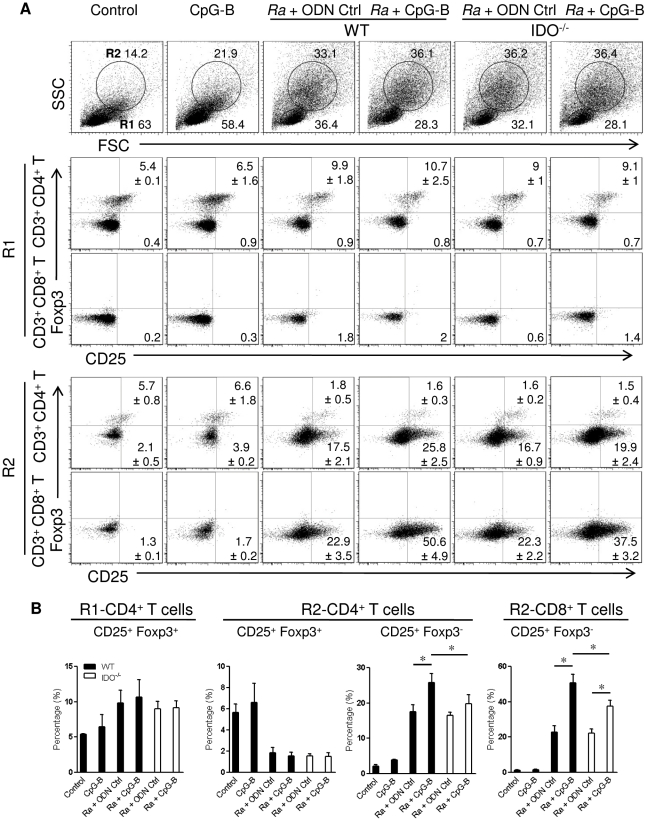
CpG-B treatment promotes T cell activation partially through IDO, without altering the frequencyof Treg cells. At day 5 post-infection, spleen cells from individual mice (4 mice per group) were stained with the indicated cell surface markers and then for intracellular Foxp3. Based on forward scatter (FSC) and side scatter (SSC) parameters, two populations were defined as quiescent cells (region 1, R1) and activated cells (region 2, R2) (A). The percentages of CD25^+^ Foxp3^+^ Treg cells and CD25^+^ Foxp3^−^ activated T cells gated in CD3^+^ CD4^+^ and CD3^+^ CD8^+^ T cells from R1 and R2 populations are shown as mean ± SD in the corner. Representative statistical data are shown from one of three independent experiments (B). * *p*<0.05.

As systemic treatment with CpGs has been reported to induce splenic Treg activation through IDO-expressing CD19^+^ DCs [14], [15], we investigated regulatory T cells in spleen by staining with anti-CD25 and anti-Foxp3 Abs. Based on forward and side scatter (FSC and SSC) parameters, we observed that rickettsial infection induced two different cell populations which could be gated as R1 (quiescent cell populations, dominantly present in the control group) and R2 (activated cell populations, mainly present in the rickettsiae-infected groups) (Figure 5A). These two cell populations were associated with splenomegaly in *R. australis*-infected mice with or without CpG-B treatment in contrast to the normal spleen size in control mice. In R1 cells (quiescent cell populations), a 2-fold increase in the frequency of Treg cells (CD4^+^ CD25^+^ Foxp3^+^) was observed in *R. australis*-infected mice, and CpG-B treatment did not affect the frequency of Treg cells in infected WT and IDO^−/−^ mice, although CpG-B alone slightly increased the frequency of Treg cells compared to controls (Figure 5A). Interestingly, in R2 cells (activated cell populations), we observed that while rickettsial infection decreased the frequencies of Treg cells in the infected mice, rickettsial infection clearly induced T cell activation (CD4^+^ and/or CD8^+^ CD25^+^ Foxp3^−^) (Figure 5A). Furthermore, treatment of infected mice with CpG-B significantly enhanced the percentages of activated T cells partially through IDO (Figure 5B). These results suggest that treatment with CpG-B does not affect the frequency of Treg cells but mainly promotes T cell activation during rickettsial infection.

**Figure 6:**
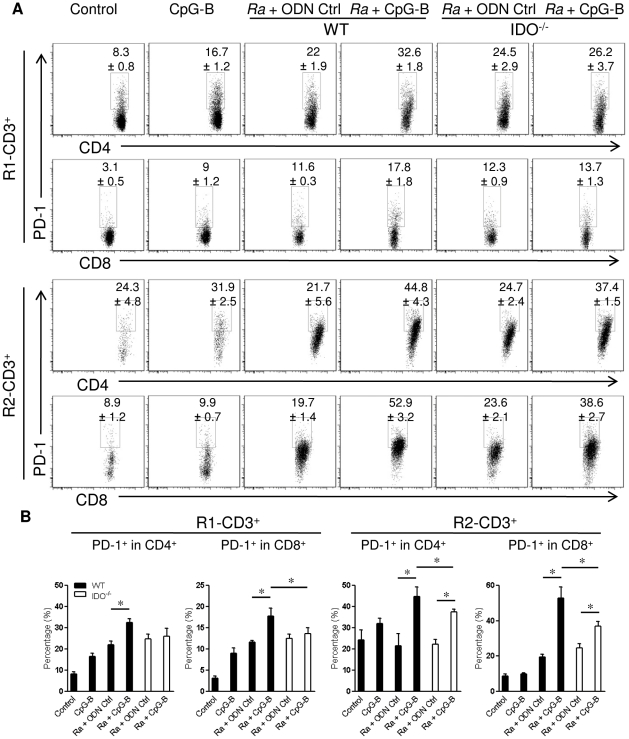
Treatment with CpG-B enhanced PD-1 expression on T cells partially through IDO. The percentages of PD-1 on CD4^+^ and CD8^+^ T cells that were gated on R1 and R2 CD3^+^ T cells (as in Figure 5) were determined by FACS at day 5 post-infection (A). The percentages of PD-1 expression in gated CD4^+^ and CD8^+^ T cells are shown as mean ± SD (B). Representative statistical data are shown from one of three independent experiments. * *p*<0.05.

Furthermore, we determined that, in the R1 and R2 cell populations, rickettsial infection induced greater PD-1 expression on CD4^+^ and CD8^+^ T cells compared to that of the control group (Figure 6). Interestingly, treatment with CpG-B dramatically increased PD-1 expression both on CD4^+^ and CD8^+^ T cells especially in the R2 population (activated cells) in *R. australis*-infected WT mice. To some extent, treatment with CpG-B only promoted PD-1 expression on R2- but not R1-T cells in *R. australis*-infected IDO^−/−^ mice (Figure 6). Therefore, treatment with CpG-B promoted PD-1 expression on quiescent and/or activated T cells partially via IDO.

### CpG-B induced cell apoptosis in *R. australis*-infected mice through IDO

**Figure 7:**
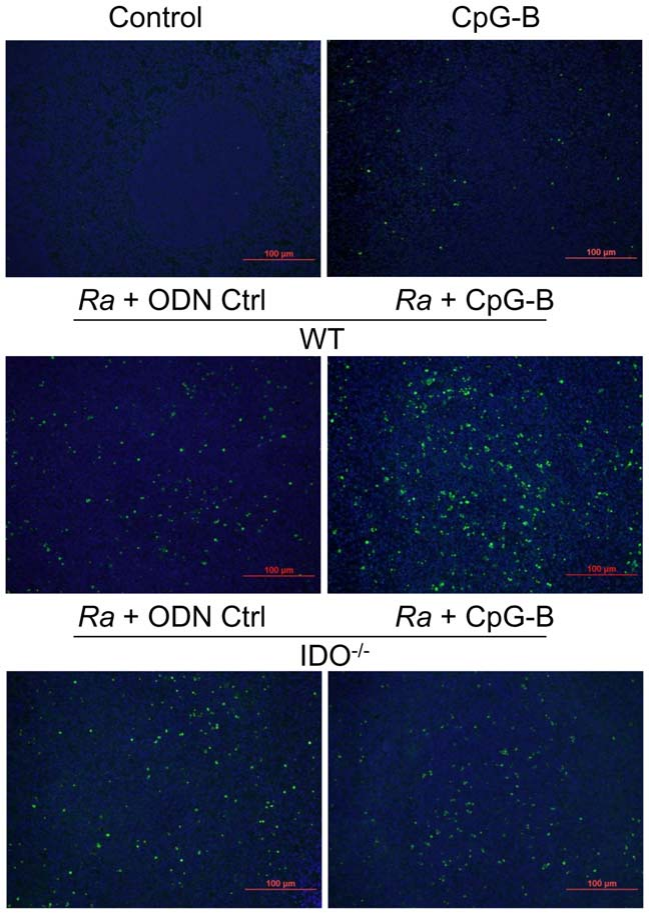
Treatment with CpG-B induced cell apoptosis through IDO. Apoptotic cells in spleen tissues at day 5 postinfection were detected by TUNEL assay (magnification ×20). Green (fluorescein) staining of apoptotic cells and blue (DAPI) staining of nuclei. Shown are the representative results from three independent experiments.

The above determinations of increased T cell activation and PD-1 expression on T cells after CpG-B treatment led us to investigate whether this T cell over-activation is related to cell death. In addition, the IDO pathway has been reported to be involved in the regulation of T cell apoptosis [22], [23]. We then performed *in situ* TUNEL assay on spleen and liver tissues from control and *R. australis*-infected mice. Histopathology revealed cellular infiltrations in *R. australis*-infected mouse liver, but there was no difference in these infiltrates between *R. australis*-infected mice with or without CpG-B treatment (Figure S1A). Similar observations were found in lung and brain tissues (data not shown). While TUNEL assay clearly showed that infection with *R. australis* induced apoptosis in mouse spleen cells, greater quantities of apoptotic cells were observed in spleen from CpG-B-treated, *R. australis*-infected WT mice than infected IDO^−/−^ mice (Figure 7). Most of the apoptotic cells were observed in the periarteriolar lymphocytic sheaths. In addition, there were a few scattered apoptotic cells observed in liver of *R. australis*-infected mice (Figure S1B). These results suggest that CpG-B promotes tissue cell apoptosis during rickettsial infection via an IDO-dependent mechanism.

### CpG-B-induced death of *R. australis*-infected mice is pDC-independent

**Figure 8:**
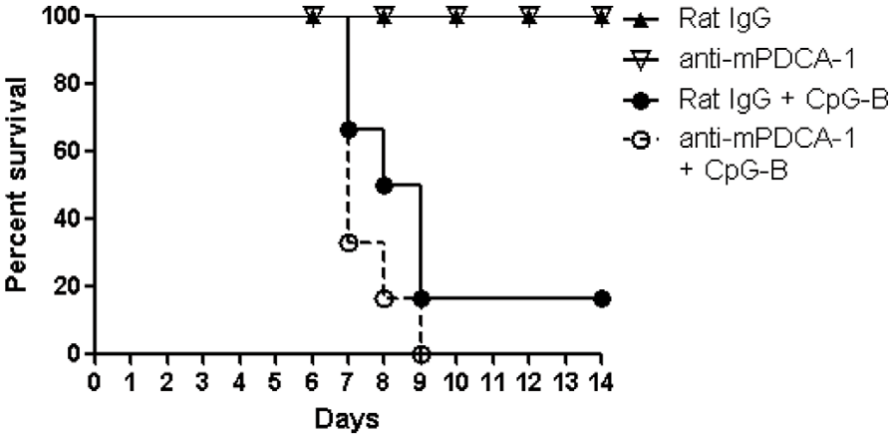
CpG-B-induced death of *R. australis*-infected mice is independent of pDC. Depletion of pDC was performed as in *Methods*. At day 2 after infection with *R. australis* (5×10^5^ pfu), 50 µg of CpG-B per mouse were injected i.v. into control or pDC-depleted mice. Mouse survival (6–8 mice per group) was monitored for 14 days.

To determine whether CpG-B-induced mouse death in rickettsial infection is mediated by induction of IDO-expressing pDCs, we performed pDC depletion by using functional grade anti-mPDCA1 antibody *in vivo*. The efficiency of pDC depletion was 80%–90% on day 7 (data not shown). Depletion of pDC did not prevent the death of *R. australis*-infected mice treated with CpG-B (Figure 8). This result strongly suggests that CpG-B-induced death of *R. australis*-infected mice is independent of pDC.

### Nitric oxide is partially involved in CpG-B-induced death of *R. australis*-infected mice

**Figure 9:**
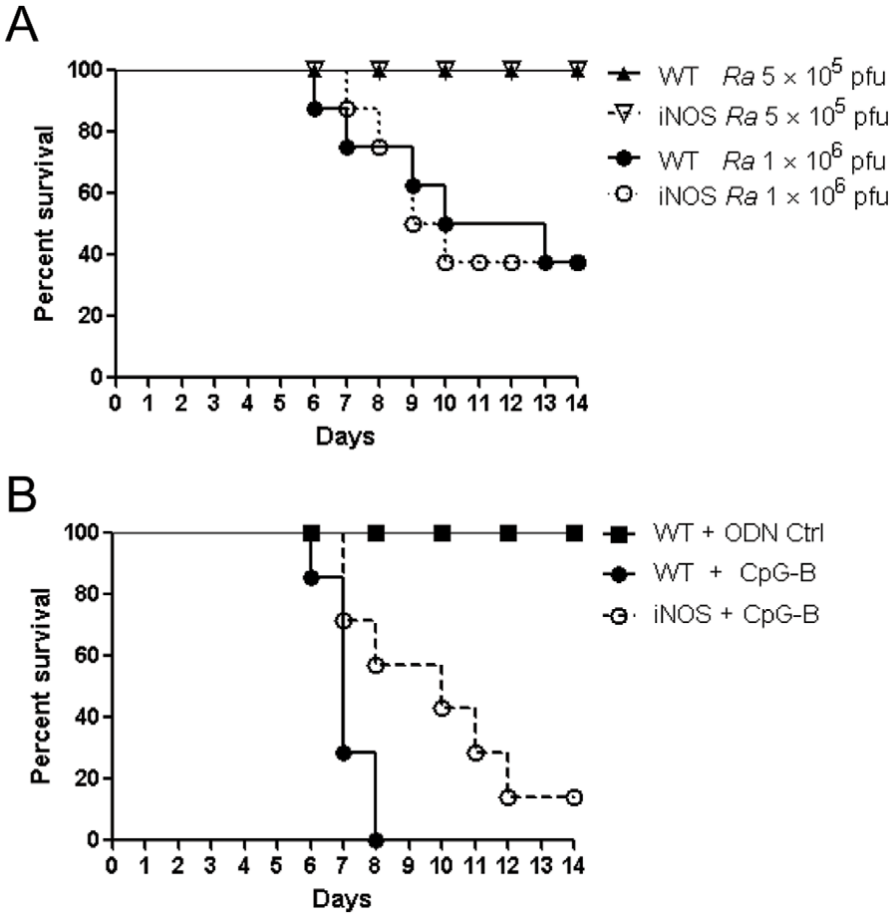
NO is partially involved in CpG-B-induced death of *R. australis*-infected mice. WT and iNOS^−/−^ mice (8 mice per group) were injected i.v. with different doses of *R. australis* (A). At day 2 after infection with 5×10^5^ pfu of *R. australis*, 50 µg of ODN control (ODN 1826 control) and CpG-B (ODN 1826) per mouse were injected i.v. into WT and iNOS^−/−^ mice (8 mice per group), respectively (B). Mouse survival was monitored for 14 days.

Since nitric oxide (NO) has been reported to play a critical role in antirickettsial activity and in CpG-mediated protection against *Listeria monocytogenes* [24], [25], [26], we further examined the role of NO in rickettsial infection in mice. Infection of WT and iNOS^−/−^ mice with a sublethal dose or a 1 LD50 dose of *R. australis* caused no major differences in mortality between WT and iNOS^−/−^ mice (Figure 9A). This result suggests that iNOS plays a dispensable role in *R. australis* infection *in vivo*. However, treatment with CpG-B at 2 days after *R. australis* infection in iNOS^−/−^ mice resulted in delayed mouse deaths compared to WT mice (Figure 9B), suggesting that NO is partially involved in CpG-B-induced death of *R. australis*-infected mice.

### Pre-treatment with CpG-B protected against lethal infection with *R. australis*

**Figure 10:**
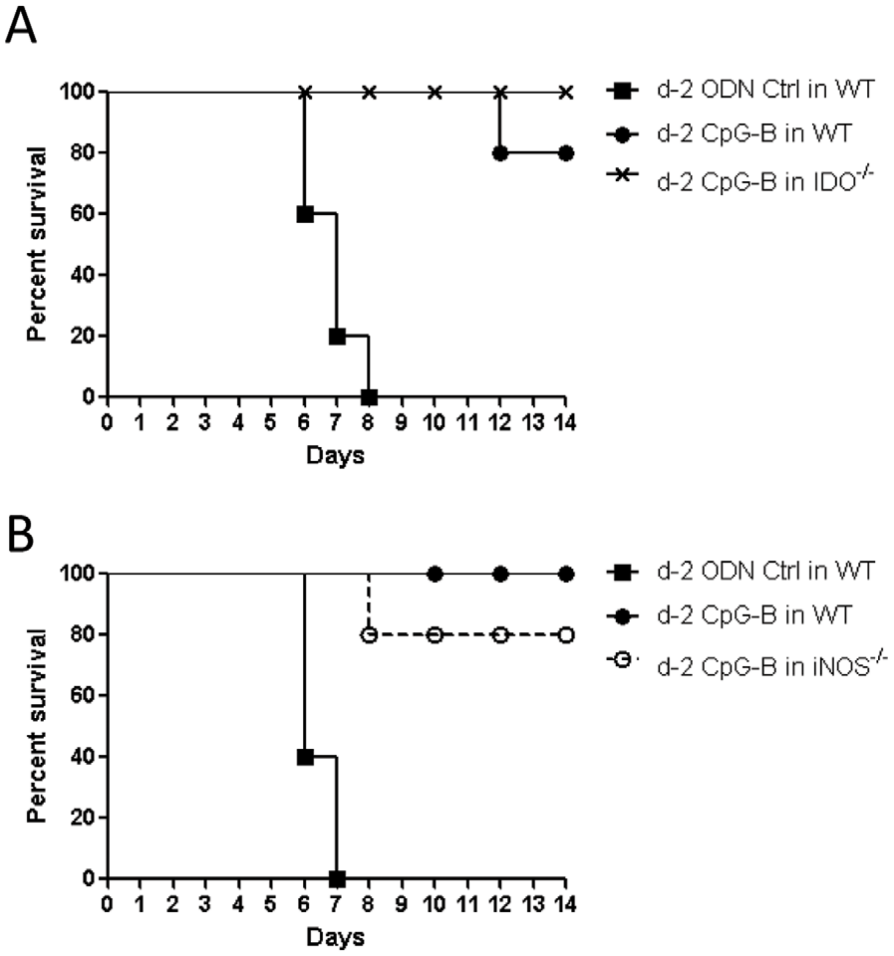
Pre-treatment with CpG-B protects against an ordinarily lethal dose of *R. australis*. 50 µg of ODN control (ODN 1826 control) and CpG-B (ODN 1826) per mouse were injected i.v. into WT and IDO^−/−^ (A) or iNOS^−/−^ mice (B) (5 mice per group) respectively. Two days later, those mice were infected with an ordinarily lethal dose of *R. australis* (2×10^6^ pfu). Mouse survival was monitored for 14 days.

CpGs have been used as a vaccine adjuvant in numerous infectious disease models [1]. Clearly, pretreatment with CpG-B at 2 days before infection efficiently protected mice against an ordinarily lethal dose of *R. australis* not only in WT mice, but also in IDO^−/−^ and iNOS^−/−^ mice (Figure 10A and 10B). Therefore, pre-treatment with CpG-B offers strong and protective immunity.

## Discussion

Our previous studies had demonstrated that CD8^+^ T cells and IFN-γ play critical roles in protection against rickettsial infection [18], [19]. In addition, TLR4-mediated innate immune responses also contribute to disease control [27]. We embarked upon determining whether use of an immunopotentiator such as CpG could enhance Th1-like immunity to increase immune resistance against lethal rickettsial infection. Indeed, pre-treatment with CpG-B before an ordinarily lethal challenge provided strong protection, which is consistent with previous findings in other infectious diseases. However, systemic treatment with CpG-B after sublethal rickettsial infection induced mouse death. The mechanistic studies suggested that the CpG-B-induced mouse death after an ordinarily sublethal infection was probably due to IDO-mediated immune regulation of the cytokine profile and T cell over-activation and apoptosis.

Activation of TLR9 leads to both proinflammatory cytokine production through a NF-κB-dependent pathway and type I IFN production through an IFN regulatory factor 7 (IRF7)-dependent pathway [28], [29]. The bifurcated signaling via TLR9 can be induced by different classes of CpGs [1]. We found that treatment with CpG-B, but not CpG-A, induced mouse death after an ordinarily sublethal rickettsial infection (Figure 1C). Clearly, systemic treatment with CpG-B in *R. australis*-infected mice enhanced the production of multiple serum cytokines/chemokines and T cell-mediated immune responses (Figure 3 and 4). Furthermore, depletion of pDC did not affect the lethal outcome of *R. australis*-infected mice when treated with CpG-B (Figure 8). These results indicate that CpG-B-induced proinflammatory cytokines, rather than type I IFNs (from pDCs), are the major contributing factors for death in *R. australis*-infected mice. Additional studies using mice deficient in the type I IFN signaling (e.g., IFNAR^−/−^ or IRF7^−/−^ mice) would verify and strengthen this conclusion.

Recent studies have identified an important role of IDO in mediating immunosuppressive effects of CpGs in the steady state [14], [16]. IDO can suppress T cell responses directly through tryptophan starvation and metabolite-mediated apoptosis [22], [23], or indirectly via induction of Treg cells by IDO-expressing pDC [14], [15], [30]. Our results showed that, although the IDO pathway is dispensable in rickettsial infection, IDO played an important role in CpG-B-induced mouse death in ordinarily sublethal *R. australis* infection since treatment with CpG-B induced very low mortality in IDO^−/−^ mice (Figure 2B). Although treatment with CpG-B alone increased the frequency of CD4^+^ CD25^+^ Foxp3^+^ Treg cells compared to controls, treatment with CpG-B did not alter the frequencies of Treg cells in *R. australis*-infected WT or IDO^−/−^ mice due to infection itself (Figure 5). However, we observed that CD4^+^ and CD8^+^ T cells from CpG-B-treated, *R. australis*-infected WT but not IDO^−/−^ mice expressed very high levels of CD25 and PD-1, which correlated with the occurrence of apoptosis in the spleen (Figures 5, 6, 7). Thus, our results indicate that in a rickettsial infection IDO may not be involved in induction of Treg cells by CpG-B. Instead, IDO may be involved in regulation of CpG-induced T cell activation and apoptosis through its metabolites and/or the PD-1/PD-L1 pathway [22], [23], [30]. Although determining whether Treg activity was altered by CpG-B via IDO during rickettsial infection requires further investigation, the T cell-mediated immune response enhanced by CpG-B is independent of IDO indicating no involvement of Treg cells (Figure 4). Furthermore, *in vivo* Treg cell depletion studies by our laboratory [31] and others suggest that Treg-mediated immune suppression may play a minor role in acute systemic infectious diseases [32], [33].

Although the immune suppressive role of IDO has been generally accepted, increasing amounts of data suggest that IDO serves more than one function in the immune system [34], [35]. Inhibition of IDO activity in the K/BxN murine rheumatoid arthritis model resulted in amelioration rather than exacerbation of the arthritic symptoms with decreased autoantibody titers, reduced levels of inflammatory cytokines, and an attenuated disease course [36]. Specifically, IDO^−/−^ mice and IDO inhibitor (1-methyl-tryptophan)-treated mice have decreased production of proinflammatory cytokines and increased survival from endotoxin shock [37], and IDO activity in bacteremic patients correlates with disease severity and case fatality [38]. Our results in this study that during rickettsial infection CpG-B differentially promoted proinflammatory cytokine production and T cell over-activation through IDO suggest a constituent role of IDO in CpG-B signaling. Thus, our study provides additional evidence that IDO may play a more complex role than its immunosuppressive role under certain disease conditions [39].

Furthermore, systemic treatment with CpGs causes lethal toxic shock within 18 h in LPS- or D-galactosamine-sensitized mice due to TNF-α-mediated fulminant apoptosis of liver cells [40], [41]. In this study, mice treated with CpG-B at day 2 post-infection began dying on day 6. Also, we did not observe massive apoptosis of hepatocytes. Nevertheless, CpG-B treatment did promote cytokine production during rickettsial infection (Figure 3), reminiscent of the systemic inflammatory response syndrome. Thus, synergistic induction of proinflammatory cytokines (such as through NF-κB activation [42]) and/or regulation of the immune response by CpGs during rickettsial infection could be responsible for the lethal outcome in mice. In addition, a recent study discovered that administration of TLR2 ligand exacerbates bacterial sepsis through neutrophil depletion by apoptosis [43]. However, we found no evidence for bone marrow neutrophil exhaustion in this study, since rickettsial infection induced similar neutrophilia in blood and bone marrow with or without CpG-B treatment, and CpG-B only slightly increased cellular recruitment of neutrophils and monocytes in the spleen (data not shown).

In contrast to previous findings of *in vitro* NO-mediated antirickettsial activity [24], [25], we found that deficiency of iNOS in iNOS^−/−^ mice did not affect rickettsial infection (Figure 9A). Further investigation is needed to determine if another NOS isoform, such as eNOS from endothelial cells (the main target cells for rickettsiae), plays an anti-rickettsial role *in vivo*. In addition, we determined that iNOS is partially involved in CpG-B-induced death of *R. australis*-infected mice (Figure 9B). Our results suggest that NO is also involved in the CpG-B signaling pathway probably through NF-κB activation [44]. On the other hand, pre-treatment with CpG-B clearly provides protection against lethal rickettsial infection in WT, IDO^−/−^ and iNOS^−/−^ mice (Figure 10), indicating that CpG-B-induced immunostimulation provided strong protection against subsequent infection by an IDO- and iNOS-independent mechanism.

CpGs have proven to be highly effective activators of innate and adaptive immunity, with applications in the prevention and treatment of infectious diseases, allergic conditions and cancer. However, post-exposure therapy with TLR9 activation is generally ineffective against rapidly progressive acute infections as compared with certain chronic infectious diseases [45], [46]. Our study highlighted the adverse effects of CpG-B when administrated post-infection in an acute infectious disease model. Another factor to be considered for immunotherapy is the route of administration, since systemic (i.v. or i.p.) application of CpGs results in T cell suppression whereas local subcutaneous (s.c.) administration induces immune stimulation [16]. A recent study has also provided evidence that i.v. and s.c. CpG administration were far less effective than peritumoral administration in cancer immunotherapy [47].

In conclusion, our study suggests that CpG exerts its immunological effects through both IDO-dependent and -independent mechanisms. Our findings for the complex and paradoxical roles for CpGs during infection have significant clinical implications. This study calls for a cautious use of CpG in immunomodulation in certain situations.

## Supporting Information

Figure S1 **Histopathologic analysis and apoptotic cell death of** ***R. australis*** **infected liver.** Control and infected liver of mice at day 5 post-infection (H & E staining, magnification ×20) (A). Apoptosis characterized by TUNEL assay in control and infected mice at day 5 post-infection (magnification ×20). Green (fluorescein) staining of apoptotic cells and blue (DAPI) staining of nuclei. Shown are the representative results from three independent experiments (B).
(TIF)
