# Supplementary figures and images for: Systemic Treatment with CpG-B after Sublethal Rickettsial Infection Induces Mouse Death through Indoleamine 2,3-Dioxygenase (IDO)
Source: PLoS One. 2012 Mar 28;7(3):e34062. doi: 10.1371/journal.pone.0034062 (PMC3314704; doi:10.1371/journal.pone.0034062)

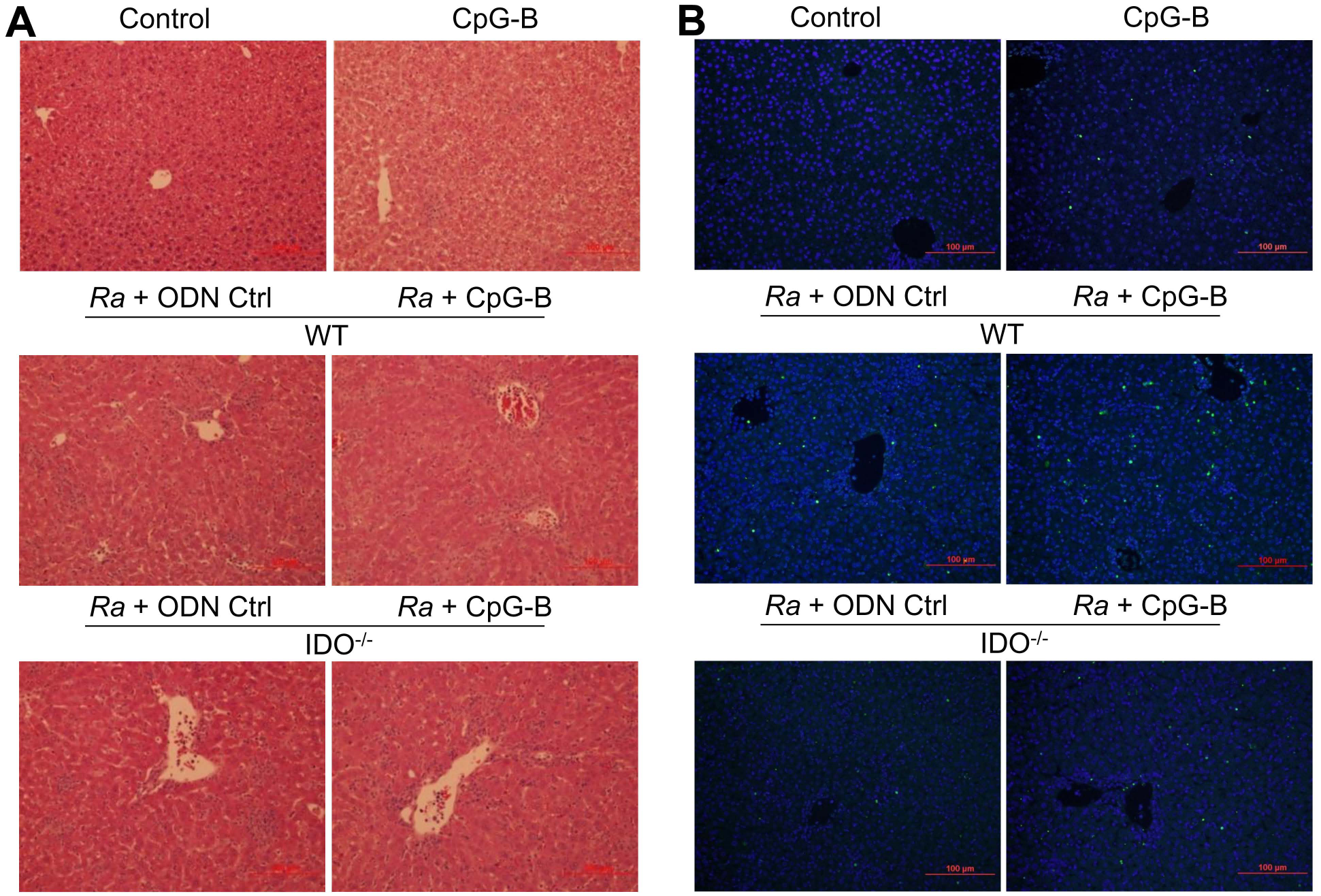

Supplement: Figure S1 — Histopathologic analysis and apoptotic cell death of R. australis infected liver. Control and infected liver of mice at day 5 post-infection (H & E staining, magnification ×20) (A). Apoptosis characterized by TUNEL assay in control and infected mice at day 5 post-infection (magnification ×20). Green (fluorescein) staining of apoptotic cells and blue (DAPI) staining of nuclei. Shown are the representative results from three independent experiments (B). (TIF) [file pone.0034062.s001.tif]
